# Early pharmacological inhibition of angiotensin-I converting enzyme activity induces obesity in adulthood

**DOI:** 10.3389/fphar.2015.00075

**Published:** 2015-04-14

**Authors:** Kely de Picoli Souza, Elton D. da Silva, Elice C. Batista, Felipe C. G. Reis, Sylvia M. A. Silva, Charlles H. M. Castro, Jaqueline Luz, Jorge L. Pesquero, Edson L. dos Santos, João B. Pesquero

**Affiliations:** ^1^School of Environmental and Biological Science, Universidade Federal da Grande DouradosDourados, Brazil; ^2^Department of Biophysics, Escola Paulista de Medicina, Universidade Federal de São PauloSão Paulo, Brazil; ^3^Department of Physiology, Escola Paulista de Medicina, Universidade Federal de São PauloSão Paulo, Brazil; ^4^Department of Rheumatology, Escola Paulista de Medicina, Universidade Federal de São PauloSão Paulo, Brazil; ^5^Department of Physiology and Biophysics, Universidade Federal de Minas GeraisBelo Horizonte, Brazil

**Keywords:** renin-angiotensin system, overweight, hyperphagia, NPY, PPAR, UCP

## Abstract

We have investigated early programming of body mass in order to understand the multifactorial etiology of obesity. Considering that the renin-angiotensin system (RAS) is expressed and functional in the white adipose tissue (WAT) and modulates its development, we reasoned whether early transitory inhibition of angiotensin-I converting enzyme activity after birth could modify late body mass development. Therefore, newborn Wistar rats were treated with enalapril (10 mg/kg of body mass) or saline, starting at the first day of life until the age of 16 days. Between days ninetieth and hundred and eightieth, a group of these animals received high fat diet (HFD). Molecular, biochemical, histological, and physiological data were collected. Enalapril treated animals presented hyperphagia, overweight, and increased serum level of triglycerides, total cholesterol and leptin, in adult life. Body composition analyses revealed higher fat mass with increased adipocyte size in these animals. Molecular analyses revealed that enalapril treatment increases neuropeptide Y (NPY) and cocaine- and amphetamine-regulated transcript (CART) gene expression in hypothalamus, fatty acid synthase (FAS), and hormone-sensitive lipase (HSL) gene expression in retroperitoneal WAT, and decreases peroxixome proliferators-activated receptor (PPAR)γ, PPARα, uncoupling protein (UCP)2, and UCP3 gene expression in WAT. The results of the current study indicate that enalapril administration during early postnatal development increases body mass, adiposity and serum lipids in adulthood associated with enhanced food intake and decreased metabolic activity in WAT, predisposing to obesity in adulthood.

## Introduction

Epidemiological studies have revealed associations between critical events in the early phases of development, such as pregnancy and suckling, and metabolic, cardiovascular diseases in the adult life (Langley-Evans, 2006, 2015). The adipose tissue is among the tissues that undergo modification in the postnatal life and its characteristics become essential to the development of obesity.

White adipose tissue (WAT) is composed of different cellular types such as preadipocytes, mature adipocytes, and pluripotent mesenchymal cells of the connective tissue, whose features can be determined by specific events in critical phases of their development (Mostyn and Symonds, 2009). Early nutritional alterations have high impact on the metabolism in adulthood, in human and animals. Children with malnutrition events present deficiency in the mechanism of fat oxidation and therefore have higher risk to develop obesity (Hoffman et al., 2000). Restriction of protein during pregnancy and lactation increases visceral adiposity in the adult and modifies the expression of genes that control the metabolism, as the expression of fatty acid synthase (FAS) enzyme in the visceral WAT, and other genes involved with adipocyte differentiation, angiogenesis and remodeling of extracellular matrix (Guan et al., 2005). Gorski et al. (2006) demonstrated that postnatal factors can overcome both genetic predisposition and prenatal factors in determining the development of adiposity, insulin sensitivity, and the brain pathways that mediate these functions. These authors have shown that obesity-prone pup cross-fostered to obesity-resistant dams remained obese but improved their insulin sensitivity in adult life (Gorski et al., 2006). In contrast, obesity-resistant pups cross-fostered to genetically obese dams showed a diet-induced increase in adiposity, reduced insulin sensitivity and associated changes in hypothalamic neuropeptide, insulin, and leptin receptors, which might have contributed to their metabolic defects (Gorski et al., 2006).

The WAT is known to produce and secrete key substances for energy metabolism control and other functions. Among these substances are the components of the renin-angiotensin system (RAS), which have been initially described only as involved in the control of arterial pressure (Cassis et al., 1988). The WAT also secretes tumor necrosis factor (TNF) α and interleukins, important hormones for signaling in the immune system and inflammatory response, respectively. In addition to the systemic effect of angiotensin II (AngII) in the regulation of blood pressure, several other functions of AngII produced by the adipocyte have been suggested: AngII has been associated with the progression of the cellular cycle of human preadipocytes (Crandall et al., 1999); AngII stimulates the production and release of prostacyclins from the adipocytes, which in turn, stimulates adipogenesis of adipose precursory cells (Saint-Marc et al., 2001); and AngII increases lipogenesis and triglycerides accumulation in 3T3-L1 cells and adipocytes (Jones et al., 1997). In accordance with these metabolic effects produced by AngII, it is also observed that rats treated with antagonist of the AngII receptor (losartan) show reduction in adipocyte size (Zorad et al., 1995). Moreover, results from Massiera et al. (2001) using angiotensinogen knockout mice submitted to high fat diet (HFD) show that AngII has local function in the development of the WAT and its cellularity. These mice have lower percentage of body fat mass, higher number of adipocytes and reduced diameter and cellular weight, reduction of the activity of the enzyme fat acid synthase and similar levels of lean mass, locomotor activity and metabolic rate when compared to the lean controls. Deficiency of other genes of the RAS, as AT1 and AT2 receptors, independently, generates animals resistant to HFD induced obesity and insulin intolerance (Kouyama et al., 2005; Yvan-Charvet et al., 2005). Yvan-Charvet et al. (2005) have shown that AT2 receptor knockout mice present normal adiposity compared with the controls, but higher number of adipocytes with reduced size. In these animals, lipid oxidation was increased, as well as the expression of genes involved in the metabolism of the lipids in the muscle as UCP3, PPARα, PPARγ, and fatty acid translocase.

Early reports have shown that angiotensin converting enzyme (ACE) inhibitors treatment is effective in decreasing body weight in animals and humans, however its mechanism of action has not been elucidated (Masuo et al., 2001; Santos et al., 2008). In agreement, Lemes et al. (2013), recently showed that the ACE DD genotype is correlated with higher serum ACE levels and is associated with arterial hypertension and with obesity related traits in boys, but not in girls, in a cohort of obese children and adolescents. In contrast, Heimann et al. (2005), have used mice harboring 1, 2, or 3 copies of the ACE gene to evaluate the quantitative role of the ACE locus on obesity. They have shown that three-copy mice fed with a high-fat diet had lower body weight and peri-epididymal adipose tissue when compared to mice harboring one- and two-ACE copies. Despite this body of knowledge linking the RAS with the adipose tissue and metabolism, the controversy remains regarding the real role of ACE activity in energy metabolism and obesity. In addition, little is known about the effects of the modulation of this system on the postnatal life. Thus, based on the rationale that environmental factors could have influence on ACE activity, especially in critical phases of the development, resulting in different phenotypic possibilities (Levy-Marchal and Czernichow, 2006), we investigated the impact of early inhibition of ACE activity on body mass development in adult life.

## Material and methods

### Animals and experimental procedures

All aspects of animal care and experimentation performed in this study agreed with ethical principles in animal research adopted by the Brazil's National Council of Animal Experimentation (CONCEA) and were approved by the Ethical Committee for Animal Research (Protocol 1015/06) from the Federal University of São Paulo. The male rats were separated after pregnancy confirmation and the female rats were maintained during pregnancy period in a temperature-controlled room (23 ± 1°C) with a 12-h light-dark cycle (lights on at 07:00 h), with free access to food and water.

**Figure 1:**
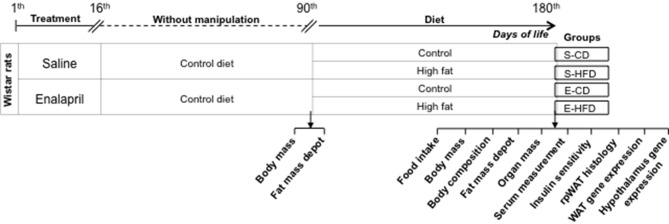
**Schematic representation of the treatments performed in the different group of animals from birth to the age of 180 days**.

As presented in the experimental schedule below (Figure 1), after birth, eight male offsprings by dam received daily either ACE inhibitor (enalapril, 10 mg/kg of BW) or saline for 16 days. Between the twenty first (weaning) and ninetieth day of life the rats were kept in standard conditions, five animals by cage, without any experimental manipulation. The rats were randomly assigned at the ninetieth day into two groups (10 animals each group). Each group received either control diet (17 kJ/g–10 kcal% fat, Research Diets, Inc.–CD) or HFD (21 kJ/g–45 kcal% fat, Research Diets, Inc.–HFD) for 3 months. For assessment of water intake and urine excretion, the rats (180-day-old) were maintained in metabolic cages for 48 h (Figure 1). After this time, the rats were sacrificed by decapitation and the blood was collected and centrifuged at 1000 g for 20 min. Samples of WAT (epididymal, retroperitoneal and mesenteric—gastrointestinal; and inguinal—subcutaneous) and hypothalamus were weighted and quickly frozen. Kidney, liver, and heart were weighted to evaluate relative mass (g of organ/100 g of body mass).

### Body composition

At the hundred and eightieth day of life the rats were anesthetized with ketamine plus xylazine (1:1, 0,1 ml/kg) and submitted to body composition analysis. The indirect body composition was analyzed by dual-energy X-ray absorptiometry (DEXA), using a hologic QDR 2000 system (software v 5.54). Lean mass and fat mass (% of total mass) were determined using the whole body (excluding head and tail).

### Serum measurements

The 180-day-old animals were sacrificed between 8:00 and 12:00 a.m. by decapitation and the blood was collected in tubes for serum leptin, glucose, total cholesterol and triglycerides measurements. Blood samples were centrifuged at 1000 g for 20 min and the sera collected were kept at −70°C until serum measurements. Sera lipoprotein and triglycerides were determined by the colorimetric method (Kit; Labtest, Minas Gerais, Brazil). Leptin (R&D systems, Inc., Minneapolis, USA) concentrations were determined by Elisa.

### Oral glucose tolerance test (OGTT)

Before this test, the diets were withdrawn for 6 h, and then 1.5g/kg body weight dose of dextrose was administered by gavage. Tail venous blood samples were collected immediately before (0 min) and at 30, 60, 120, and 180 min after dextrose gavage. The blood glucose level of these samples was analyzed using a glucometer (Precision QID, Abbott Laboratories Medisense Products, Bedford, MA, USA). The data obtained were plotted, and the area under the curve (AUC) was calculated.

### Intravenous insulin tolerance test (ivITT)

Rats were deprived of food for about 2 h and anesthetized with ketamine plus xylazine (1:1, 0,1 ml/kg), and then received an intravenous injection of regular insulin (0.75 U/kg body weight). Glucose levels were measured on samples obtained from the tail vein using a glucometer (Precision QID, Medisense, São Paulo, SP, Brazil) immediately before (0 min) and at 4, 8, 12, and 16 min after insulin injection. The corresponding 4–16 min values were used to calculate the rate constant for plasma glucose disappearance (kITT).

### Histological analysis

Retroperitoneal adipose tissue of animal at 180 days of life was fixed in 10% (v/v) formaldehyde/PBS and embedded in paraffin, sliced (4 μm) and staining with hematoxylin and eosin. Morphometric parameters (number of cells per field and major axis) were determined by computer-assisted image analysis using software written in the macro language Quips (Quantimet Interactive Programming System) of the Leica Qwin package (Leica Microsystems Ltd., Cambridge, United Kingdom).

### Gene expression analyses

Total RNA from the WAT epididymal depot and hypothalamus were extracted from control and enalapril treated animals at 180 days of life using the TRIzol^®^Reagent (Invitrogen, USA) according to the manufacture's recommendation. One microgram of RNA was reverse transcribed using Moloney Murine Leukemia Virus reverse transcriptase (Invitrogen). First-strand cDNA synthesized from total RNA with random hexamers was used as the template for each reaction. The iCycler iQ Real Time PCR Detection System (Bio-Rad) was used for the signal detection, and the PCR was performed using QuantiTec SYBR Green PCR (QUIAGEN GmbH, Germany) using 300 nmol/L of each primer. For standardization and quantification, mice β-actin was amplified simultaneously. The primer sequences employed for the amplification are shown in Table 1. PCR conditions were 50°C for 2 min, 95°C for 10 min, followed by 45 cycles at 95°C for 15 s, and 60°C for 60 s. Fluorescence was detected at the end of every extension phase (72°C). Data generated from SYBR Green were analyzed according (Livak and Schmittgen, 2001). Calculation of the fold change in specific gene each was relative to the β-actin endogenous control using 2^−ΔCt^.

**Table 1:** **Primer sequences**.

**Transcript**	**Gene symbol**	**Forward Primer**	**Reverse Primer**
Neuropeptide Y	NPY	GAGGACGCGCCAGCAGAGG	GTCTCAGGGCTGGATCTCTTGC
Cocaine- and amphetamine-regulated transcript	CART	GTGCCACGAGAAGGAGCTG	CACATGGGGACTTGGCCGTAC
Proopiomelanocortin	POMC	TGCCAGGACCTCACCACGG	GTGACCCATGACGTACTTCCGG
Agouti related protein	AgRP	GATGATCTGCTGCAGAAGGC	TTGAAGAAGCGGCAGTAGCACG
Peroxisome proliferator-activated receptor alpha	PPARα	TCCACGAAGCCTACCTGAAG	GAACTCTCGGGTGATGAAGC
Peroxisome proliferator-activated receptor gamma	PPARγ	AACATCCCCAACTTCAGCAG	GGAAGAGGTACTGGCTGTCG
Uncoupling protein 2	UCP2	AATGTTGCCCGAAATGCC	CAATGACGGTGGTGCAGAA
Uncoupling protein 3	UCP3	ATGGATGCCTACAGAACCAT	CTGGGCCACCATCCTCAGCA
Fatty acid synthase	FAS	CTTGGGTGCCGATTACAACC	GCCCTCCCGTACACTCACTC
Hormone-sensitive lipase	HSL	CCCATAAGACCCCATTGCCTG	CTGCCTCAGACACACTCCTG
ß-actin	ß-actin	AGAGGGAAATCGTGCGTGAC	CCATAGTGATGACCTGTCCGT

### Statistical analysis

The data were subjected to unpaired *t*-test to compare animals treated with saline (*n* = 20) and enalapril (*n* = 20). When submitted to different diet [control (*n* = 10) or HFD (*n* = 10)] the animals were compared into the same treatment. Data were expressed as means ± standard error of means (SEM). Differences were considered significant at *p* < 0.05.

## Results

### Effects of postnatal ACE inhibition on body mass development

**Figure 2:**
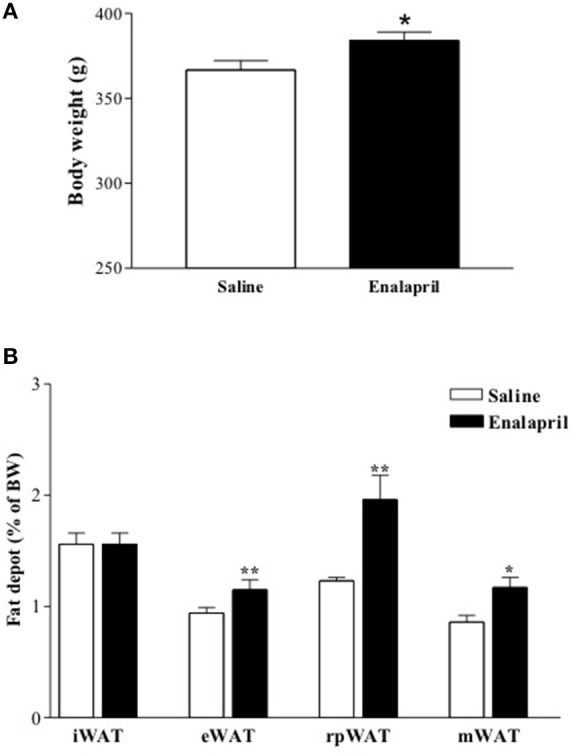
**(A)** Body and **(B)** fat depot mass of 90 days old rats treated with saline or enalapril (10 mg/Kg of BW) during the first 16 days after birth.^*^p < 0.05, ^**^p < 0.01 vs. saline treated animals. Data are shown as mean + SEM, *n* = 10 rats each group. Inguinal (iWAT), epididymal (eWAT), retroperitoneal (rpWAT) and mesenteric (mWAT) fat depots mass.

In order to evaluate the impact of postnatal ACE inhibition on late body weight evolution, newborn rats were treated with enalapril (10 mg/kg) or saline for 16 days after birth. Body weight was measured in the animals at birth and once a week until the ninetieth day of life. It was observed on the sixteenth day, end of the treatment, that the body weight was similar between both groups of animals (25 ± 1 vs. 23 ± 1 g of BW, saline and enalapril), however on the ninetieth day it was significantly higher in the enalapril treated group (Figure 2A). Except for the inguinal WAT, all the other depots increased in enalapril treated rats when compared to saline group (Figure 2B).

### Impact of HFD in the body weight of rats treated with enalapril

**Figure 3:**
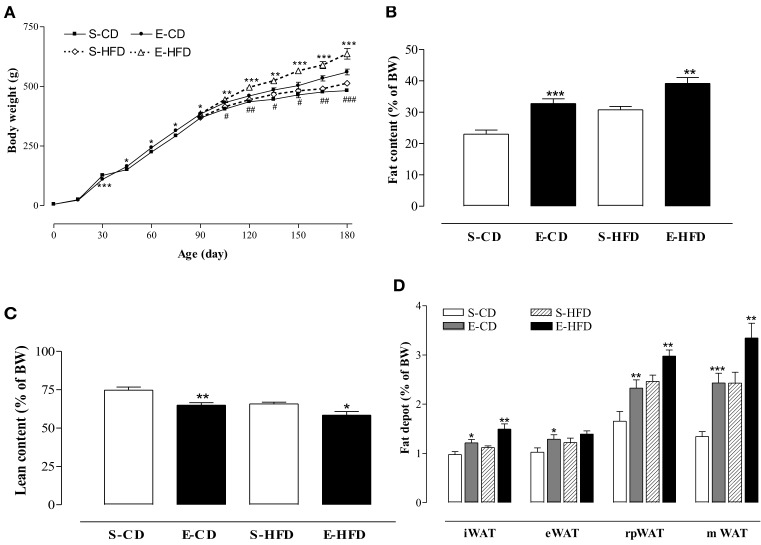
**Effect of high fat diet in the mass and body composition after postnatal enalapril treatment**. **(A)** Body weight (BW), evolution of adult rats treated with control (CD), and high fat (HFD) diet from the ninetieth day until the hundred and eightieth day of life. **(B)** Percentage of fat, **(C)** lean body mass, and **(D)** inguinal (i), epididimal (e), retroperitoneal (rp), and mesenteric (m) fat depots of white adipose tissue (WAT) of rats treated with saline (S) or enalapril (E) for 16 days after birth. ^*^p < 0.05, ^**^p < 0.01, ^***^p < 0.001 vs. control group (animals fed with the same diet). ^##^*p* < 0.01, ^###^*p* < 0.001 E-HFD vs. E-CD group. Data are shown as mean + SEM, *n* = 10 rats each group.

To analyze the sensitivity of the iACE treated animals to a hypercaloric regimen, as well as the evolution of the effects of enalapril postnatal treatment, on the ninetieth day the rats were subdivided in four groups and fed with control (CD) or high fat (HFD) diet for 3 months. The rats treated with enalapril kept their overweight at the hundred and eightieth day (Figure 3A), showing an elevated Lee index and food intake and hyperphagia (Table 2). In addition, enalapril treated animals fed with HFD had the highest increase in body weight at 180 days, showing a 24% overweight when compared to control group (Figure 3A).

**Table 2:** **Anthropometric data (body weight and Lee index), food intake, organs weight (kidney, liver and heart), and the index of water consumption/urine excretion of rats treated with saline (S) or enalapril (E) and fed from day ninetieth to hundred and eightieth after birth with control (CD) or high fat (HFD) diet**.

**Groups**	**S-CD**	**E-CD**	**S-HFD**	**E-HFD**
Body Weight gain (g)	126 ± 5	179 ± 12^***^	158 ± 5	235 ± 26^**^
Lee Index	6.3 ± 0.1	6.8 ± 0.1^**^	6.5 ± 0.2	7.2 ± 0.2^*^
Food intake (kJ/day)	284 ± 11	328 ± 5^**^	305 ± 4	338 ± 9^**^
Rate water/urine (g/g)	2.3 ± 0.5	1.6 ± 0.1	1.8 ± 0.6	2.0 ± 0.4
Kidney (% of BW)	0.18 ± 0.02	0.16 ± 0.01	0.14 ± 0.01	0.15 ± 0.01
Liver (% of BW)	2.8 ± 0.1	2.8 ± 0.1	2.4 ± 0.1	2.4 ± 0.1
Heart (% of BW)	0.17 ± 0.02	0.15 ± 0.01	0.14 ± 0.01	0.14 ± 0.01

Data are expressed as mean ± SEM of 10 animals/group.

* p < 0.05,

** p < 0.01, and

*** p < 0.001 when compared S and E group in same diet.

Lee index = rate body weight (g) and by nasoanal lengths (mm).

The transient treatment with iACE was able of modify the body composition of the animals in adulthood. As shown in Figures 3B,C, in the enalapril treated animals the fat mass was increased in both diets whereas the lean mass was decreased. The inguinal (iWAT), epididymal (eWAT), retroperitoneal (rpWAT), and mesenteric (mWAT) fat depots mass, normalized by body weight, increased in enalapril treated animals in both diets in 180 days old rats, except eWAT in HFD (Figure 3D). On the other hand, the iACE did not provoke any weight difference in the heart, liver, and kidney (Table 2). No difference in rate of water intake by urine excretion was observed between the groups (Table 2).

### Insulin sensitivity and glucose tolerance

**Figure 4:**
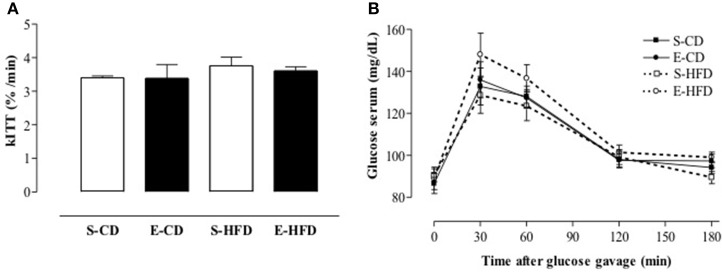
**Insulin sensitivity after postnatal enalapril treatment**. Animals treated with saline (S) or enalapril (E) during the first 16 days after birth and fed from day ninetieth to hundred and eightieth after birth with control (CD) or high fat diet (HFD) were submitted to **(A)** intravenous insulin test (0.75 U/kg body weight) and **(B)** oral glucose tolerance test (1.5 g of glucose/Kg of BW by gavage). Data are shown as mean + SEM, *n* = 10 rats each group.

In order to evaluate the metabolic consequences of overweight and elevated adiposity in the iACE treated rats, intravenous insulin tolerance test (ivITT), and oral glucose tolerance test (OGTT) were performed (AUC: 521 ± 18, 490 ± 16, 574 ± 28, 542 ± 13, S-CD, S-HFD, E-CD, E-HFD respectively; *p* > 0.05 when groups in the same diet were compared). Insulin and glucose tolerance were similar between the groups (Figures 4A,B).

### Lipid profile, glucose, and leptin levels of rats treated with enalapril

Accordingly to increased body weight and fat mass, enalapril treated animals also showed elevated triglycerides, total cholesterol and leptin level when compared to saline treated animals in the same diet (Table 3). No difference was observed in serum glucose levels between the groups in both diets (Table 3).

**Table 3:** **Lipid profile, glucose, and leptin levels in the serum of animals treated with iACE (E–10 mg/kg of BW) or saline (S) for 16 days after birth and fed from day ninetieth to hundred and eightieth after birth with control (CD) or high fat (HFD) diet**.

**Groups**	**S-CD**	**E-CD**	**S-HFD**	**E-HFD**
Triglycerides (mg/dL)	156 ± 18	256 ± 41^*^	95 ± 5	163 ± 20^**^
Total cholesterol (mg/dL)	116 ± 12	154 ± 10^*^	98 ± 2	131 ± 6^***^
Glucose serum (mg/dL)	106 ± 3	111 ± 10	108 ± 3	116 ± 4
Leptin (ng/mL)	7 ± 1	11 ± 2^*^	9 ± 1	17 ± 4^*^

Data are expressed as mean ± SEM of 10 animals/group.

* p < 0.05,

** p < 0.01, and

*** p < 0.001 when compared to S and E group in same diet.

### Histological analysis of WAT in adult life

**Figure 5:**
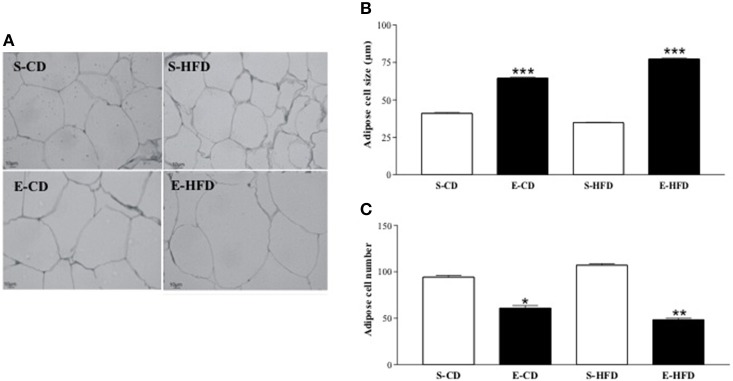
**Adipocyte size and adipocyte number after postnatal enalapril treatment. (A)** Histological analyses (20x magnification) of retroperitoneal WAT, **(B)** Adipocyte size, and **(C)** number of rats treated with saline (S) or enalapril (E) during 16 days after birth and fed with control (CD) or high fat (HFD) diets between day ninetieth and hundred and eightieth. ^*^p < 0.05, ^**^p < 0.01, ^***^p < 0.001 vs. control group (animals fed with the same diet). Data represent mean ± SEM, *n* = 5 animals each group.

Histological analysis of the retroperitoneal WAT at 180 days of life showed an increase in adipocyte size and a reduced number of adipose cells in the enalapril treated animals fed with both diets when compared to the respective control group (Figures 5A–C).

### Gene expression in rats after postnatal enalapril treatment

**Figure 6:**
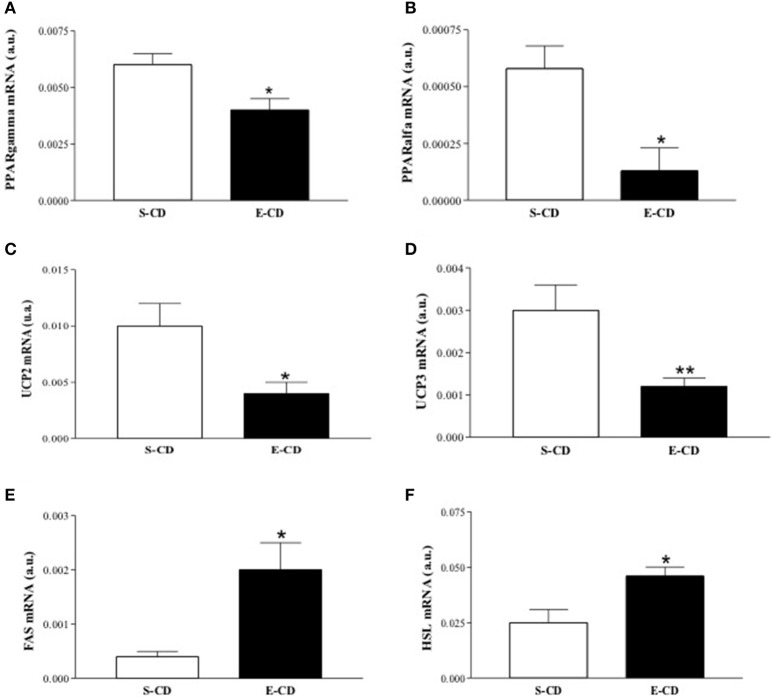
**Expression of lipolytic and lipogenic genes in the retroperitoneal WAT after postnatal treatment with enalapril**. Animals were treated with saline (S) or enalapril (E–10 mg/kg BW) during the first 16 days after birth and treated for 90 days with control diet. WAT was collected and mRNA expression of **(A)** PPARγ, **(B)** PPARα, **(C)** UCP2, **(D)** UCP3, **(E)** FAS, and **(F)** HSL was analyzed at 180 days of life. Data represent mean ± SEM, *n* = 5 animals each group. ^*^p < 0.05 and ^**^p < 0.01 when compared to the saline group.

**Figure 7:**
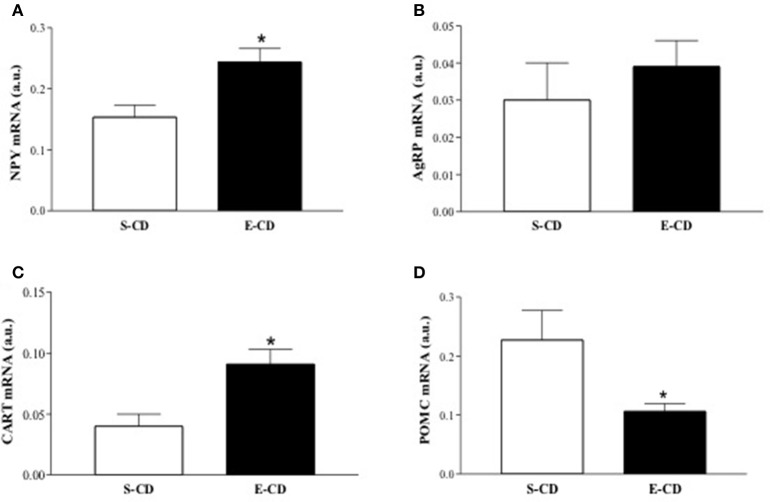
**Expression of hypothalamic genes after postnatal treatment with enalapril**. Animals were treated with saline (S) or enalapril (E – 10 mg/kg BW) during the first 16 days after birth and treated for 90 days with control diet. Hypothalamus was collected and mRNA expression of **(A)** NPY, **(B)** AgRP, **(C)** CART, and **(D)** POMC was analyzed at 180 days of life. Data are present as mean ± SEM, *n* = 5 animals each group. ^*^p < 0.05 when compared to the saline group.

To elucidate the molecular basis of functional and histological modifications observed in enalapril treated animals, we investigated metabolism-related gene expression in rpWAT and hypothalamus. In the rpWAT it was observed a reduced gene expression of both PPARs (α and γ) and UCPs (2 and 3) isoforms and elevation of FAS and HSL mRNA in enalapril treated animals compared with control group (Figures 6A–F). Gene expression analyses in hypothalamus reveal NPY and CART mRNA increase and proopiomelanocortin (POMC) mRNA decrease in enalapril treated animals compared with control group (Figures 7A,C,D). No difference was observed in agouti related protein (AgRP) mRNA expression (Figure 7B).

## Discussion

In this study, we showed that early postnatal treatment with an ACE inhibitor, enalapril, applied to newborn rats during the first 16 days after birth is responsible for a programming in metabolism, favoring hyperphagia and WAT accumulation. This altered metabolism results in overweight in adult life with increased body adiposity, serum lipid profile, and leptin, despite of preserved insulin sensitivity and serum glucose.

The reduced ACE activity with enalapril treatment affects at least the function of two relevant physiological systems, RAS and kallikrein-kinin system. Classically, these systems have been linked to cardiovascular and inflammatory processes especially in adult phase. However, both systems also have pivotal roles during postnatal development and their activation can change the maturation of the adipose tissue (Martins et al., 2008; Mori et al., 2008). Obesity resistance phenotype has been generated upon deletion of many receptors in these systems (Yvan-Charvet et al., 2005; Mori et al., 2008) as well as of the AngII precursor molecule angiotensinogen (Massiera et al., 2001), which corroborates the physiological relevance of these systems in the regulation of metabolic activity and body mass maintenance. Metabolic alterations were also obtained with the insertion of three copies of the ACE gene into the mouse genome, although divergent data have been observed. In this case the genetic augmentation of ACE activity triggers a phenotype of decreased body fat and mass under hyperlipidic diet (Heimann et al., 2005), in opposition to other studies that point positive correlation between ACE activity and obesity (Davidson et al., 2011). These apparently contrasting findings could be due to other functions attributed to ACE not related to AngII production, like the signaling activity of the enzyme (Kohlstedt et al., 2005; Guimaraes et al., 2011).

In the present study the activity of the RAS was inhibited only during a specific postnatal phase and it was possible to verify that the role of AngII in differentiation in adipose tissue (Ailhaud et al., 2002) for the generation of adipogenic precursors can be age-dependent. In our study, AngII blockade in early postnatal life was able to modify adult WAT cellularity and metabolism. Early postnatal enalapril treated rats show reduced hyperplasia and elevated hypertrophy of retroperitoneal adipocytes. PPARγ is highly expressed in adipose tissue, and is a transcription factor important to adipocyte differentiation and regulation of genes involved in lipid utilization and storage (Powell et al., 2007; Lee et al., 2011). PPAR agonists contribute to prevent obesity in adult life and other associated diseases by decreasing adiposity and increasing the expression of antioxidant and lipolytic genes (Willson et al., 2001; Bassaganya-Riera et al., 2011). Many *in vitro* studies (Albrektsen et al., 2002) indicate that PPARγ activation agonists stimulate adipocyte differentiation through the activation of a variety of adipogenic genes. PPARγ is essentially required for the formation, differentiation, and survival of WAT (Rosen et al., 1999; Imai et al., 2004). Enalapril treated rats show decreased PPARα and PPARγ gene expression in retroperitoneal WAT, which is the key molecular mechanism to explain the reduced number of adipose cells. The activation of PPARγ causes structural remodeling of adipocytes in adult WAT that is characterized by increased number of smaller adipocytes (Okuno et al., 1998). The origin of these smaller adipocytes is postulated to be the amplified differentiation of residential preadipocytes or the active division of adipocytes themselves (Okuno et al., 1998). Moreover, PPARα and PPARγ are nuclear regulators of WAT, regulating UCP2 e UCP3 gene expression (Villarroya et al., 2007).

UCP2 and UCP3 mRNA are positively correlated with PPARs gene expression, which could express of body mass in enalapril treated animals. UCP2 is expressed in many tissues and has been considered a significant factor for basal metabolism (Gjedde et al., 2010). Chronic treatment with rosiglitazone, a thiazolidinedione capable of activating PPARγ, has been reported to involve an induction in UCP3 mRNA levels in WAT (Matsuda et al., 1998; Emilsson et al., 2000). Additionally, the revised data by Cecil et al. (2006), about the role of PPARγ gene polymorphisms in the energy balance and food intake, suggest that the role of PPARγ is varied and complex, influencing the speed of fat deposition and growth early in life, with potential impact in the control of energy intake and appetite regulation. Gene expression of the enzymes FAS and HSL are augmented in the enalapril treated rats. These alterations contribute to the increase in triglycerides and total cholesterol serum levels shown by the enalapril treated rats.

Our data of transitory administration of enalapril in postnatal life are opposite to the findings of Basso et al. (2007) and Santos et al. (2008) obtained by chronic enalapril treatment in rats after weaning, which led to decreased body mass and leptin serum level. The elevation in the body weight gain observed in this work indicates a higher susceptibility to overweight in the neonatal animals treated with ACE inhibitors, which could reflect an AngII age-dependent effect on the metabolism during development. Despite their common use as first-line therapy for selected patients with chronic hypertension and for the prevention of diabetic nephropathy, ACE inhibitors are not indicated for women during pregnancy and lactation due to their excretion into breast milk (Ghanem and Movahed, 2008). In addition to their effects on the metabolism, Mecawi et al. (2009) have also shown that ACE inhibition during prenatal and neonatal periods affects behavioral responses in adult offspring rats, suggesting that ACE is required for the development of neural systems that are associated with adult anxiety and nociceptive behavioral responses.

The increased food intake is in agreement with elevated NPY gene expression and reduced POMC gene expression in the enalapril treated rats. Together, an increased expression of an orexigen neuropeptide and a decreased expression of anorexigen neuropeptide trigger hyperphagia, and the decreased metabolic profile of the WAT in enalapril treated rats might be responsible for the overweight observed in animals. It is possible that leptin, although increased in enalapril treated animals, might fail to reduce NPY expression as occurs in obese humans due to resistance to its actions (Friedman, 2011). CART, an anorexigenic neuroptide, responsible for the elevation in energy expenditure in excess energy state, can be induced by leptin levels elevation (Dominguez, 2006). Although enalapril treated rats have presented elevated CART gene expression, this effect was not sufficient to counterbalance the other modification in the opposite metabolic direction.

Although the amount of leptin, fat mass, total cholesterol and triglycerides were increased in enalapril treated rats, we could not establish a correlation between insulin resistance and higher body mass in this study. Other counter-regulators such as adiponectin and TNFα, which had similar expression in saline and enalapril treated rats (data not shown), have collaborated for the maintenance of insulin sensitivity.

**Figure 8:**
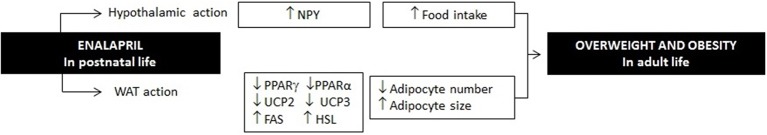
**Enalapril administration in rats in postnatal life increases hypothalamic NPY gene expression, decreases expression of PPAR in the WAT and leads to long term consequences in metabolism, WAT cellularity and obesity**.

In conclusion, altogether our data show that postnatal early pharmacological inhibition of ACE modifies the development of WAT and hypothalamus, leading to hyperphagia, hyperlipidemia and overweight in adulthood (Figure 8).

### Conflict of interest statement

The Editor Claudio M. Costa-Neto declares that, despite having collaborated with author João B. Pesquero, the review process was handled objectively and no conflict of interest exists. The authors declare that the research was conducted in the absence of any commercial or financial relationships that could be construed as a potential conflict of interest.
